# Effect of cyclin-dependent kinase 7 silencing on cisplatin sensitivity in endometrial carcinoma cells

**DOI:** 10.3892/mmr.2014.2980

**Published:** 2014-11-19

**Authors:** WEN-XIN LIU, XIANG-YU LIU, HU YU, YING CHEN, QUAN HAO

**Affiliations:** Department of Gynecologic Neoplasms, Tianjin Medical University Cancer Institute and Hospital, Tianjin 300060, P.R. China

**Keywords:** endometrial carcinoma, CDK7, RNA interference, cisplatin, chemotherapy

## Abstract

The aim of the present study was to determine the effect of cyclin-dependent kinase 7 (CDK7) silencing on the sensitivity of the HEC-1-A endometrial carcinoma cell line to cisplatin [cis-dichlorodiammineplatinum (II), or DDP]. Four CDK7 siRNA fragments were designed and synthesized based on the gene sequence of CDK7 and transfected into HEC-1-A cells. The RNA interference of the fragments was confirmed by semi-quantitative polymerase chain reaction (PCR) and western blot analyses. The CDK7-423 siRNA fragment exhibited the most marked silencing of CDK-7 (>70%), and was chosen for the subsequent experiments in HEC-1-A endometrial carcinoma cells. The sensitivity of the cells to a chemotherapeutic agent (cisplatin) was determined before and after transfection of the siRNA, using a MTT cytotoxicity assay, flow cytometry and Hoechst/propidium iodide (PI) double-staining immunofluorescence microscopy. The results of the MTT cytotoxicity assay showed that the half maximal inhibitory concentration of cisplatin was reduced from 45.12 μg/ml to 3.200 μg/ml following the inhibition of CDK7 expression levels, indicating a significantly increased cytotoxicity in the treated cells (P<0.05). The flow cytometry analysis showed that the mean rate of apoptosis in the CDK7 low-expression group was 37.57%, which was significantly higher than the rate in the parental cells (11.66%) (P<0.05). Hoechst/PI co-immunofluorescence microscopy revealed that the number of apoptotic bodies in the CDK7 low-expression HEC-1-A cells was significantly increased as compared with the parental cells. Downregulation of CDK7 expression levels in HEC-1-A endometrial carcinoma cells via the transfection of CDK7 siRNA may significantly enhance cancer cell sensitivity to cisplatin chemotherapy and increasing apoptosis. CDK7 is a novel promising treatment for endometrial carcinoma that requires further in-depth study.

## Introduction

Endometrial carcinoma is a common malignancy of the female genital tract. The incidence of endometrial carcinoma has increased in recent years, and it has become the most common gynecological malignancy in a number of European and American countries (1). By ruling out interference from various confounding factors with laser capture microdissection technology, cyclin-dependent kinase (CDK) 7 has been identified as a differentially expressed gene that is highly correlated with endometrial carcinoma (2,3). As a basic structural component of CDK-activating kinase (CAK), CDK7 has a key role in the cell cycle. Abnormal expression of CDK7 disturbs the balance of the cell cycle and promotes DNA replication and mitosis, resulting in abnormal cell growth, replication, and differentiation; abnormalities that are closely associated with the occurrence and development of tumors. Additional research following these studies revealed that the expression levels of CDK7 were lowest in normal endometrium, increased during endometrial hyperplasia and peaked in endometrial carcinoma tissues (4).

Based on preliminary research, the present study used siRNA technology to silence CDK7 expression in the HEC-1-A endometrial carcinoma cell line. Changes in the cisplatin [cis-dichlorodiammineplatinum (II), or DDP] sensitivity of cells were detected by MTT cytotoxicity assay in addition to flow cytometry and Hoechst/propidium iodide (PI) co-immunofluorescence microscopy. The aims of the present study were to clarify the association between CDK7 expression levels and the sensitivity of HEC-1-A cells to cisplatin, and to reveal the mechanism underlying chemotherapy resistance in endometrial carcinoma cells, providing a novel theoretical basis for the improvement of chemotherapy efficacy. To the best of our knowledge, no similar studies have been performed either domestically or internationally.

## Materials and methods

### Human endometrial carcinoma cell culture

HEC-1-A cells were purchased from the Tianjin Institute of Hematology (Tianjin, China) and were routinely cultured in Dulbecco’s modified Eagle’s medium (DMEM; Gibco-BRL, Carlsbad, CA, USA) containing 10% fetal bovine serum (FBS). The cells were maintained in a humidified incubator at 37°C with 5% CO_2_.

### Design and synthesis of CDK-7 siRNA fragments

GenePharma siRNA designing software was used to design the siRNA fragments (GenePharma, Shanghai, China). Based on the gene sequence of human CDK7, four different siRNA fragments, including CDK7-1, CDK7-2, CDK7-423 and CDK7-910, were designed and synthesized by Shanghai UNJA Biotechnology, Ltd. (Shanghai, China).

### Transfection of siRNA

HEC-1-A cells were grown to 80–90% confluence and subsequently inoculated onto 6-well plates at a density of 8×10^5^ cells/well. Once they had been mixed thoroughly, the cells were cultured at 37°C in a 5% CO_2_ incubator for 24 h. The cells were divided into six groups: CDK7-1, CDK7-2, CDK7-423, CDK7-910, the negative control (with siRNA constructed from unrelated sequences), and the blank control group (normal cultured cells). Opti-MEM serum-free culture medium (250 μl; Gibco-BRL) and 100 pmol siRNA were added to a 1.5 ml Eppendorf (EP) tube, while 250 μl of Opti-MEM and 5 μl of Lipofectamine™ 2000 (Gibco-BRL) were added into a second EP tube. Following gentle mixing, the solutions were placed at room temperature for 5 min, gently mixed again and incubated at room temperature for 20 min. Subsequently, the culture medium was removed, and 1.5 μl of Opti-MEM was added to each well. The transfection mixture was added dropwise to 6-well plates, and the cells were incubated for 4–6 h. Following incubation, the transfection solution was discarded, and 500 μl of DMEM culture medium containing 10% FBS was added. The cells were cultured at 37°C in 5% CO_2_ for 48 h and harvested for RNA extraction or western blot analysis. The transfection efficiency = the number of GFP-labeled cells/total number of cells × 100, where GFP is green fluorescence protein.

### Reverse transcription-quantitative polymerase chain reaction (RT-qPCR) analysis

The total RNA in the transfected cells was extracted using TRIzol^®^ (Invitrogen, Carlsbad, CA, USA) following the manufacturer’s instructions. cDNA was then synthesized and the qPCR used the following primers: CDK7 upstream, 5′-AGGATGTATGGTGTAGGTGTGGA-3′, and downstream, 5′-AAGATGTGATGCAAAGGTATTCC-3′ (amplification length, 221 bp) and GAPDH upstream, 5′-AGAAGGCTGGGGCTCATTTC-3, ′ and downstream 5′-AGGGGCCATCCACAGTCTTC-3′ (amplification length, 220 bp). The primers were designed using the Primer Premier 5.0 software (PREMIER Biosoft, Palo Alto, CA, USA) and were synthesized by Shanghai Sangon Biological Technology Co., Ltd. (Shanghai, China). The cycling conditions for the reverse transcription were as follows: 70°C for 5 min, followed by immediate cooling on ice; 42°C for 30 min; and 85°C for 10 min for reaction termination, followed by immediate cooling on ice. cDNA synthesis was conducted using a MuLV reverse transcriptase kit (Applied Biosystems, Foster City, CA, USA) according to the manufacturer’s instruction. The cDNA reaction solution was used as a template for the subsequent step. qPCR was performed using an Applied Biosystems 7500 Real-Time PCR Machine and data were analyzed using Step One Software v.2.1 (Applied Biosystems). GAPDH was used as an internal normalization control. The results are represented as the fold change in gene expression relative to that of GAPDH (2^−ΔΔCt^). The primers and probes chosen from Roche’s UPL system were as follows: CDK7 (accession no: NM_000077.4) with UPL probe #34, and GAPDH (accession no: NM_0000194.4) with UPL probe #73. The reaction conditions for the quantitative PCR were as follows: 95°C for 2 min; followed by 40 cycles of 95°C for 20 sec, 60°C for 30 sec and 72°C for 30 sec; and finally a 72°C extension for 10 min.

### Western blot analysis

A total of 100 μl cell lysis buffer (Beyotime, Shanghai, China). was added to each well of cells. The lysate was then transferred into a centrifuge tube and heated to 100°C for 5 min. Following cooling on ice, the sample was centrifuged at 12,000 × g for 10 min to remove any insoluble precipitate. Subsequently, the harvested sample was separated using 10% SDS-PAGE (Beijing Biyutian Co., Ltd., Beijing, China). Following electrophoresis, the sample was transferred onto a polyvinylidene fluoride membrane (Shanghai ShuoGuang Technology Co., Ltd., Shanghai, China) and blocked using 5% skim milk (Gibco-BRL). The membrane was incubated with anti-CDK7 antibodies (1:500, Abcam, Cambridge, UK) at 4°C overnight. Subsequently, the cells were incubated with horseradish peroxidase-labeled rabbit anti-goat IgG (ZSGB-BIO, Beijing, China) at an appropriate dilution at room temperature for 2 h. Chemiluminescence detection was performed using the enhanced chemiluminescence (ECL) reagent (Roche, Basel, Switzerland) and exposed to ECL X-ray films (Roche). After being developed and fixed, images of the films were captured using a gel imaging analysis system (BioRad Laboratories, Hercules, CA, USA ). The results were analyzed using the Gel-Pro-Analyzer (Media Cybernetics, Georgia, MD, USA). GADPH was used as the internal control and the experiments were repeated three times.

### MTT cytotoxicity assay

HEC-1-A cells at the logarithmic growth phase were transfected with CDK7-423. After 48 h, the cells were dissociated, harvested and inoculated onto a 96-well plate, with 7,000–8,000 cells/well. Once the cells attached, they were treated with 0.4, 2.0, 10.0, 50.0 or 250.0 μg/ml cisplatin (Qilu Pharmaceutical Co., Shandong, China) and incubated at 37°C in 5% CO_2_ for 48 h. Subsequently, 10 μl of MTT (5 mg/ml; Shanghai Yuanye Biological Technology Co., Ltd., Shanghai, China.) was added to each well, and the plates were incubated at 37°C for 4 h. The culture plates were removed from the incubator and centrifuged at 550 × g for 5 min. The supernatant was discarded and 100 μl dimethylsulfoxide (Shanghai Yuanye Biological Technology Co., Ltd.) was added to each well to terminate the reaction and dissolve the purple/blue formazan. The mixtures were vortexed and each well’s absorbance value was detected using a μQuant microplate reader (Bio-Tek Instruments Inc., Winooski, VT, USA) at a wavelength of 490 nm wavelength. The inhibition rates of the cisplatin-treated HEC-1-A cells were calculated prior to and following CDK7 siRNA transfection. The calculated rates were then used for curve fitting and half maximal inhibitory concentration (IC_50_) calculations.

### Analysis of cell cycle and detection of apoptosis rate using flow cytometry

HEC-1-A cells were counted prior to and after CDK7 siRNA transfection and adjusted to a final concentration of 1×10^6^ cells/ml. Subsequently, 2 ml of cells were inoculated onto a 6-well plate. Following treatment with cisplatin (10 μg/ml) for 48 h, the cells were dissociated, collected and stained with PI. The cells were detected and analyzed using an Elite flow cytometer (Coulter Cytometry, Inc., Hialeah, FL, USA).

### Observation of nucleus morphological changes using immunofluorescence microscopy

HEC-1-A cells were counted prior to and after CDK7 siRNA transfection, and the cell concentration was adjusted to 7–8×10^4^ per ml. Subsequently, 1 ml of cells was inoculated onto a 24-well plate and treated with cisplatin (10 μg/ml) for 48 h. A staining solution of Hoechst 33258 in phosphate-buffered saline was added and incubated at 37°C for 15 min. PI dissolved in PBS (10 g/ml) was added at room temperature for 15 min to cause a reaction. Images of the cells were captured under a fluorescence microscope (Leica, Mukwonago, WI, USA).

### Statistical methods

The χ^2^ test was performed using SPSS version 13.0 software (SPSS, Inc., Chicago, IL, USA). Comparisons between the means of two groups were performed using an independent groups t-test. Comparisons of the means among multiple samples were performed using single-factor analysis of variance (ANOVA). An analysis of the time- and dose-dependent responses was performed using an ANOVA of factorial design. P<0.05 was considered to indicate a statistically significant difference.

## Results

### Transfection efficiency of CDK7 siRNA increases in a time-dependent manner

**Figure 1:**
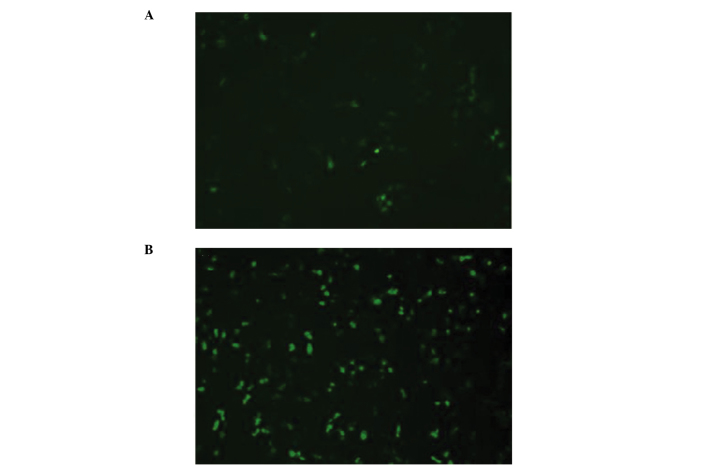
Green fluorescent protein detection of cyclin-dependent kinase 7 siRNA transfection efficiency at (A) 24 and (B) 48 h post-transfection. Magnification, ×100

CDK7 siRNA was labeled with GFP and transfected into HEC-1-A cells. The results showed that the transfection efficiency at 48 h was significantly higher than that at 24 h, reaching ~70% (Fig. 1).

### CDK7 siRNA inhibits CDK7 expression levels in HEC-1-A cells

**Figure 2:**
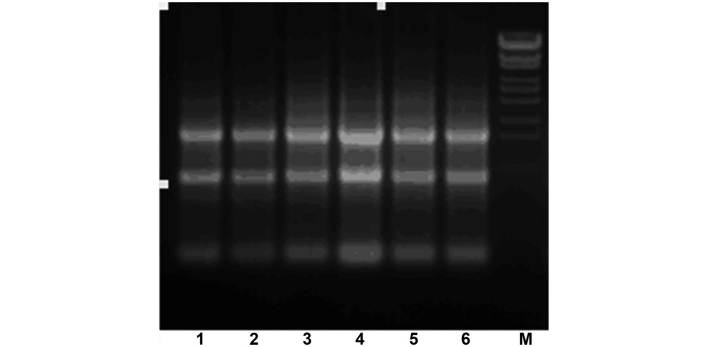
Analysis of RNA integrity, obtained from cells transfected with CDK7 siRNA. Lane 1, CDK7-1 siRNA; lane 2, CDK7-2 siRNA; lane 3, CDK7-423 siRNA; lane 4, CDK7-910 siRNA; lane 5, negative control; lane 6, blank control; and lane M. CDK7, cyclin-dependent kinase 7; M, marker.

**Figure 3:**
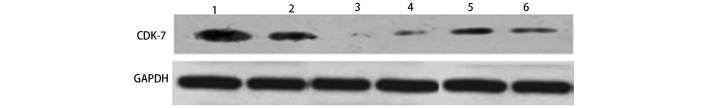
Western blotting analysis for CDK7, in cells treated with four siRNAs, a negative control and blank control groups. Lane 1, CDK7-1 siRNA; lane 2, CDK7-2 siRNA; lane 3, CDK7-423 siRNA; lane 4, CDK7-910 siRNA; lane 5, negative control; and lane 6, blank control. CDK7, cyclin-dependent kinase 7.

The total RNA in CDK7-transfected cells was extracted using the TRIzol^®^ method. The obtained RNA was dissolved in diethyl phosphorocyanidate (DEPC)-treated water, and the integrity of the RNA was analyzed using agarose gel electrophoresis (Fig. 2). The PCR amplification curves, standard curves and dissociation curves of CDK7 and GADPH were satisfactory. A comparison revealed that the interference effect of CDK7-423 (group 3) was the strongest (Table I). The results of the western blotting revealed that the siRNA interference in each group reduced the CDK7 protein expression levels. The interference effect of CDK7-423 was the strongest, which corresponded to the results of the western blotting (Fig. 3).

### Suppression of CDK7 expression in HEC-1-A cells induces significantly higher cisplatin cytotoxicity

**Figure 4:**
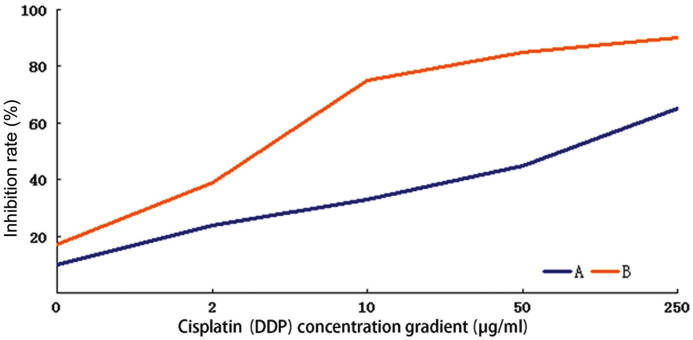
Curves displaying the 48h inhibition rate of HEC-1-A following administration with different concentrations of cisplatin. A, before transfection; B, after transfection.

The inhibition rates induced by 48-h treatment with different concentrations of cisplatin were calculated in the HEC-1-A cells before and after CDK7-423 siRNA transfection, and a fitted curve was obtained for the determination of IC_50_. The IC_50_ of cisplatin was 45.122 μg/ml in the parental HEC-1-A cells, which reduced to 3.200 μg/ml following transfection with CDK7-423 siRNA. The concentration gradient of cisplatin used in this experiment and the corresponding inhibition rates are shown in Fig. 4. The 48-h cisplatin treatment induced significantly higher cytotoxicity in the HEC-1-A cells with inhibited CDK7 expression, compared with that observed in the parental cells (P<0.05).

### CDK7 knockdown by siRNA increases the apoptosis rates in cisplatin-treated HEC-1-A cells

**Figure 5:**
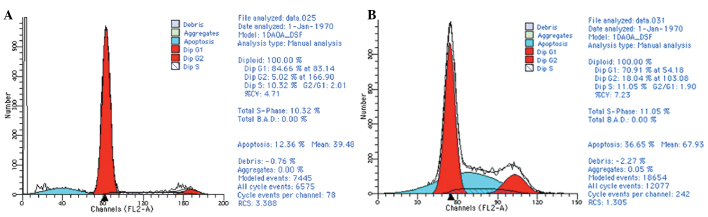
Analysis of cell apoptosis by flow cytometry in (A) HEC-1-A cells or (B) HEC-1-A cells transfected with CDK7-423 siRNA, both treated with 10 μg/ml cisplatin.

Following the 48-h cisplatin treatment, at a final concentration of 10 μg/ml, the mean apoptosis rates were 11.66% in the parental HEC-1-A cells and 37.57% in the cells transfected with CDK7-423 siRNA (P<0.05) (Fig. 5).

### Cisplatin treatment significantly increases the number of apoptotic bodies in HEC-1-A cells with low CDK7 expression levels

**Figure 6:**
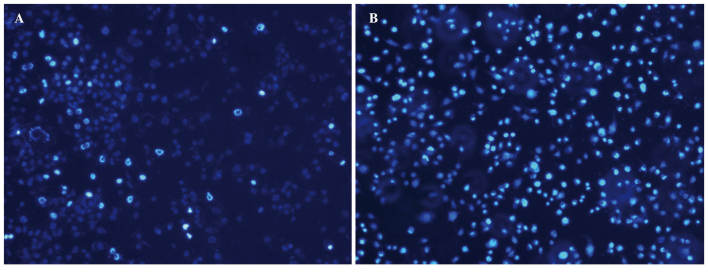
Fluorescence microscopy analysis of (A) HEC-1-A and (B) HEC-1-A cells transfected with CDK7-423 siRNA transfection, both treated with 10 μg/ml cisplatin. Magnification, ×100.

Following treatment with 10 μg/ml cisplatin for 48 h, observations under the immunofluorescence microscope showed that the number of apoptotic bodies (bright aggregates or snowflake-shaped fluorescent spots that are the characteristic spots of apoptotic nuclei, caused by chromatin condensation) in HEC-1-A cells transfected with CDK7-423 siRNA significantly increased, when compared with those observed in the parental cells (P<0.05) (Fig. 6).

## Discussion

CDKs belong to the serine/threonine kinase family. Currently, nine CDK family members (CDK1-CDK9) have been discovered in mammals. By binding to different cyclins to form complexes, these CDKs directly or indirectly act on the different phases of the cell cycle to maintain normal cell growth, differentiation and proliferation. Disturbances in the cell cycle may result in persistent cell growth and eventually tumor occurrence (5). Unlike other CDK family members (CDKs 1–4 and 6) that are directly involved in the cell cycle, CDK7 primarily participates in the regulatory processes of the cell cycle. As an important component of CAK, CDK7 has a pivotal role in cell cycle regulation. CAK is a complex consisting of three subunits: CDK7, cyclin H and MAT1. Of these subunits, CDK7 is the catalytic subunit and cyclin H is the regulatory subunit. CDK7 phosphorylation activates CAK, resulting in the phosphorylation and activation of the CDK molecules that bind to mitotic-type cyclins (including, cyclin A and B), thereby stimulating cells to enter the M phase from the G_2_ phase. In addition, CDK7 phosphorylation-induced CAK activation phosphorylates and activates CDK molecules, which bind to G_1_-phase cyclins (including, cyclin D and E) and promote cells to enter the S phase from the G_1_ phase, thus encouraging cell division and proliferation (6). Additionally, this complex is an important component of the basic transcription factor TFIIH. TFIIH catalyzes the phosphorylation of the large subunit of RNA polymerase II to trigger the transcription process. It also participates in type II transcription and nucleotide excision repair to prolong the transcription phase; as a result, genes that participate in cell division and proliferation are expressed, again promoting cell proliferation (7).

Due to its regulatory effect on the activity of other CDKs, CAK has a key role in the process of cell cycle regulation. As an important component of CAK, CDK7 is a promising therapeutic target for a variety of anti-carcinoma chemotherapeutic regimens. Previous studies have reported that the silencing of CDK7 expression via a number of methods suppresses the growth and proliferation of liver carcinoma, lymphoma, leukemia, intestinal carcinoma and breast cancer cells (8–12). Using the structure of CDK7 as a starting point, Liu *et al* (13) established a molecular docking model of CDK7 inhibitors and synthesized a novel CDK7 inhibitor; their results showed that this novel compound had inhibitory effects on HL60 acute promyelocytic leukemia cells, KB nasopharyngeal carcinoma cells, SMMC-7721 liver carcinoma cells, HCT-116 colon adenocarcinoma cells and A549 lung carcinoma cells. Therefore, CDK7 was hypothesized to be a novel target for a variety of anti-carcinoma drug treatments.

Among the various clinical treatment measures, chemotherapy has an important role in the comprehensive treatment of endometrial carcinoma. Postoperative chemotherapy is essential for the eradication of residual tumor cells. Chemotherapy is becoming the first-line treatment for advanced-stage cancer patients with small residual lesions and early-stage high-risk cancer patients; it is the primary treatment method for advanced and recurrent endometrial carcinomas. Platinum-based drugs (such as cisplatin and carboplatin) are the most widely used chemotherapy drugs in endometrial carcinoma; however, the efficacy of endometrial carcinoma chemotherapy is not satisfactory. Previous studies have reported that theefficacy of cisplatin alone is ~30%; and whilst combined chemotherapy may have increased efficacy, the toxic side-effects increase accordingly (14–16). Chemotherapy resistance causes cancer treatments to be ineffective, resulting in enormous physical, psychological and economic losses to patients. Therefore, developing methods to increase chemotherapy sensitivity (to agents including cisplatin) and overcome drug resistance has become a research hotspot in the clinical treatment of endometrial carcinoma.

Yang (17) used gene chip technology to screen the differentially expressed genes in cisplatin-resistant lung adenocarcinoma cells and found that CDK7 was highly expressed, suggesting that CDK7 is associated with cisplatin resistance in lung carcinomas. RNA interference technology was used to specifically silence CDK7 and observe the effect of CDK7 downregulation on the biological characteristics of cisplatin-resistant A549/CDDP human lung carcinoma cells. The results revealed that in lung adenocarcinoma, CDK7 is involved in the development of cisplatin resistance. In addition to its effect on cell cycle regulation, CDK7 may also mediate cisplatin resistance via the drug resistance-associated protein pathway. However, RNA interference may partially reverse the CDK7-mediated drug resistance in lung adenocarcinoma cells. Therefore, it is possible that CDK7 may be used as a gene therapy target for chemotherapy resistance in lung adenocarcinoma.

Based on the previous studies described, the current study focused on CDK7 as a research target. To the best of our knowledge, no studies on silencing CDK7 in endometrial carcinoma cells using siRNA technology have been reported, either domestically or internationally. In the present study, four different siRNA fragments were designed based on the sequence of the CDK7 gene. These were successfully transfected into the endometrial carcinoma cell line HEC-1-A.

The results of RT-qPCR and western blotting indicated that each type of interfering RNA suppressed the levels of CDK7 protein expression to varying degrees. The RNA interference mediated by CDK7-423 was the strongest, inhibiting >70% of the protein expression compared with that of the controls. To reveal the association between CDK7 and platinum resistance in endometrial carcinoma cells, CDK7-423 was selected to specifically reduce CDK7 expression in HEC-1-A endometrial carcinoma cells, and the MTT cytotoxicity assay, flow cytometry and Hoechst/PI double-staining immunofluorescence microscopy were used to detect changes in cisplatin sensitivity. The results showed that following 48-h cisplatin treatment, the IC_50_ of cisplatin was 45.122 μg/ml in the parental HEC-1-A cells, while it was only 3.200 μg/ml in the CDK7 low-expression group, indicating a statistically significant higher cytotoxicity in the cells with low CDK7 expression than in the parental cells (P<0.05). Following the 48-h cisplatin (10 μg/ml) treatment, the average apoptosis rate was 11.66% in parental HEC-1-A cells, which increased to 37.57% in the CDK low-expression group (P<0.05). Compared with the parental HEC-1-A cell group, the number of apoptotic cells in the CDK7 low-expression group was significantly increased, as observed under a fluorescence microscope. These results indicate that following the suppression of CDK7 expression levels in endometrial carcinoma cells, the sensitivity of the cells to the chemotherapy drug cisplatin was significantly increased. Thus, high CDK7 expression may be one of the mechanisms underlying the resistance of endometrial carcinoma cells to platinum-based chemotherapy.

In conclusion, the results of the present study may provide novel ideas and a theoretical basis to improve the clinical efficacy of chemotherapy and to reverse chemotherapy resistance. Further in-depth studies using CDK7 as a target for endometrial carcinoma treatment should be performed.

## Figures and Tables

**Table I:** Ct values of CDK7 and GAPDH

Identifier	CDK7 (Ct)	GAPDH (Ct)
1	17.57±0.21	11.77±0.19
2	18.14±0.35	12.20±0.08
3	18.74±0.32	12.06±0.12
4	18.45±0.18	12.31±0.21
5	17.34±0.19	12.65±0.24
6	17.62±0.38	12.82±0.33

1, CDK7-1; 2, CDK7-2; 3, CDK7-423; 4, CDK7-910; 5, negative control; 6, blank control. Ct, threshold cycle; CDK7, cyclin-dependent kinase 7.
